# Hard and Soft Tissue Management of a Localized Alveolar Ridge Atrophy with Autogenous Sources and Biomaterials: A Challenging Clinical Case

**DOI:** 10.1155/2016/8468763

**Published:** 2016-09-21

**Authors:** C. Maiorana, D. Andreoni, P. P. Poli

**Affiliations:** ^1^Oral Surgery and Center for Maxillary Atrophies, Fondazione IRCCS Cà Granda Ospedale Maggiore Policlinico, School of Dentistry, University of Milan, Milan, Italy; ^2^Private Practice, Milan, Italy; ^3^Center for Maxillary Atrophies, Fondazione IRCCS Cà Granda Ospedale Maggiore Policlinico, University of Milan, Milan, Italy

## Abstract

Particularly in the premaxillary area, the stability of hard and soft tissues plays a pivotal role in the success of the rehabilitation from both a functional and aesthetic aspect. The present case report describes the clinical management of a localized alveolar ridge atrophy in the area of the upper right canine associated with a thin gingival biotype with a lack of keratinized tissue. An autogenous bone block harvested from the chin associated with heterologous bone particles was used to replace the missing bone, allowing for a prosthetic driven implant placement. Soft tissues deficiency was corrected by means of a combined epithelialized and subepithelial connective tissue graft. The 3-year clinical and radiological follow-up demonstrated symmetric gingival levels of the upper canines, with physiological peri-implant probing depths and bone loss. Thus, the use of autogenous tissues combined with biomaterials might be considered a reliable technique in case of highly aesthetic demanding cases.

## 1. Introduction 

The long-term success of an implant-supported rehabilitation is strictly influenced by both the density and volume of available bone and the quality of soft tissues at the implant site. Particularly in case of ridge atrophies in the premaxillary region, the creation of an optimal bone support to dental implants is mandatory to guarantee an ideal functional and aesthetic outcome. This aim cannot be achieved in a satisfactory way without even considering the quality of the soft tissues surrounding an implant, especially in thin gingival biotypes.

Such a sophisticated and multiple-staged approach basically provides for several surgical steps and one or two temporary prostheses before going to the final restoration. Further, an overall treatment time of about 18–24 months might be considered.

Alveolar bone reconstruction can be obtained by means of different surgical procedures, including autogenous blocks harvested from intraoral or extraoral donor sites, guided bone regeneration (GBR), ridge splitting or expansion techniques, and distraction osteogenesis [1]. The choice of the technique mainly depends on both size and extension of the defect, on the clinical history of the tooth loss as well as the patient's compliance and expectations.

The purpose of the present paper was to present the surgical and prosthetic management of a paradigmatic clinical case characterized by a localized atrophy of the upper jaw subsequent to a failed previous reconstruction due to an impacted canine extraction. An intraoral bone block combined with bone substitutes was used to regenerate the missing bone, in association with soft tissue grafts to manage the soft tissues deficiency.

## 2. Case Presentation

**Figure 1:**
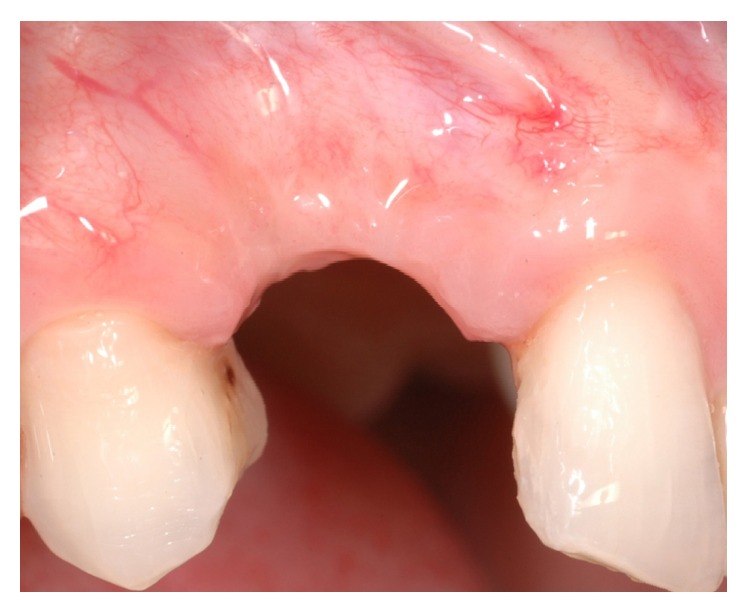
Clinical view of the lateral aspect of the edentulous site.

**Figure 2:**
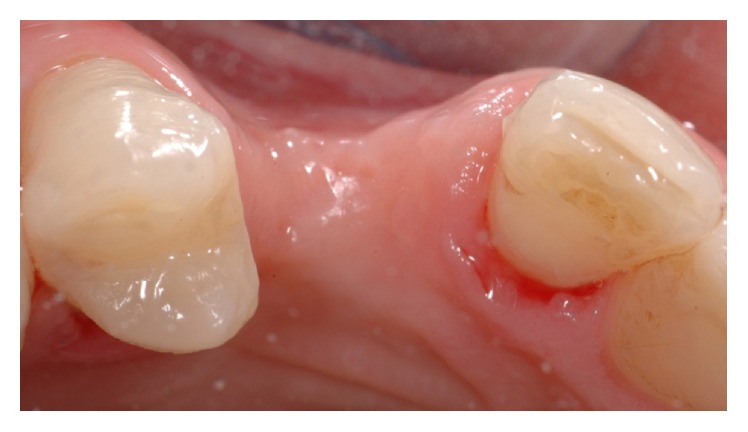
Clinical view of the occlusal aspect of the edentulous ridge.

**Figure 3:**
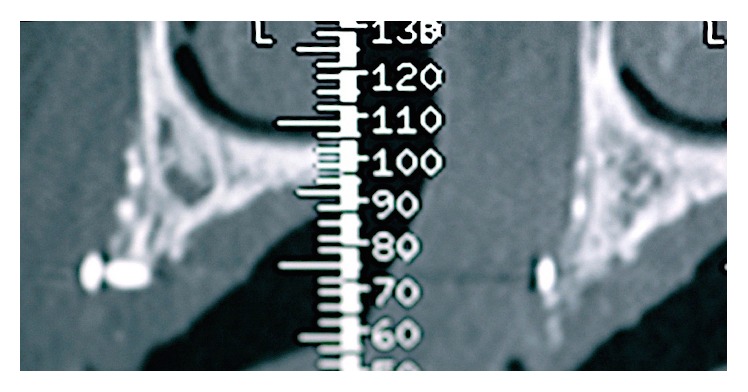
Cross-sectional CBCT scan showing the residual graft material and the cortical screw more coronally.

A 42-year-old female attended the office to investigate a localized ridge atrophy in the area of the upper right canine (Figure 1). The patient was in good general health, with no history of systemic diseases, drug allergies, and smoking habits. Upon clinical examination, the patient was found to have a horizontal bone loss with a minimal vertical component and a coronal ridge width of 2 mm (Figure 2). With respect to the soft tissues, the site was characterized by a thin gingival biotype with no keratinized mucosa. Further, the patient presented a skeletal class I deep bite with a history of an impacted canine extraction and a bone regeneration procedure carried out two years earlier, followed by infection of the graft itself. Radiographic examination, carried out with a Cone Beam Computed Tomography (CBCT) scan, confirmed the clinical evaluation and showed remnants of a non-well-defined graft consisting of few granules of a radiopaque material and a transcortical screw (Figure 3). The patient feared to be treated with a conventional bridge and was seeking for an implant treatment and an aesthetic solution. Contextually, the patient complained about a dental apicoectomy performed in the lower incisors area, followed by a mucosal fistula occurred one year later.

The proposed treatment plan consisted of an orthodontic treatment in order to correct the deep bite and obtain a teeth alignment, followed by a bone and soft tissues reconstruction to place implants in a prosthetically driven position, two provisional crowns, and a gold ceramic crown. The patient refused to undergo the orthodontic treatment; hence only the rehabilitation of the canine area was chosen.

**Figure 4:**
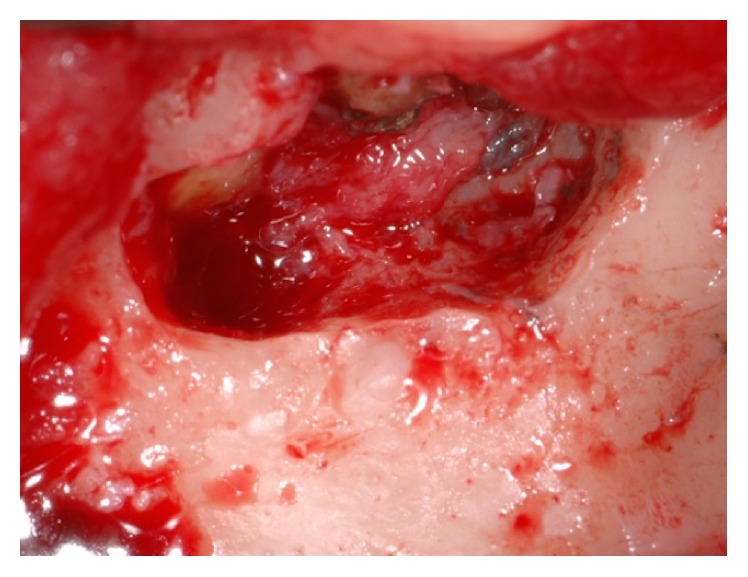
Apicoectomy surgery performed in the symphyseal area.

**Figure 5:**
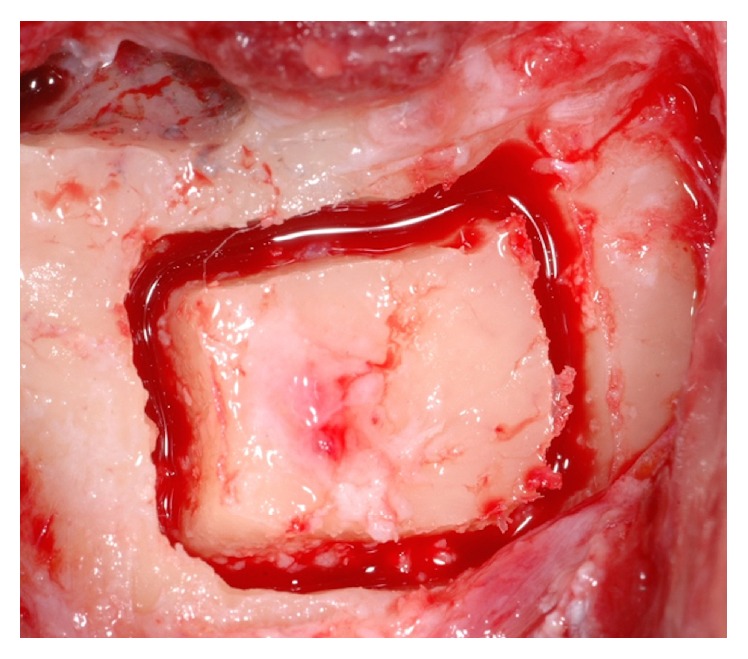
Harvesting procedure of a corticocancellous bone block in the mandibular symphysis.

The first step consisted in the reconstruction of the bone defect by means of an intraoral corticocancellous block graft harvested from the chin area, taking advantage of the simultaneous treatment of the apicoectomy area in the lower incisors region. Before the surgery, the patient was provided with a full-mouth disinfection session. One day before the appointed surgical session, the patient was instructed to start with an antibiotic therapy consisting of amoxicillin clavulanate (Augmentin®, GlaxoSmithKline S.p.A., Verona, Italy) 1 g twice daily for six days. On the day of the surgery, mepivacaine 2% with epinephrine 1 : 100.000 (Carbocaina, AstraZeneca S.p.A., Milan, Italy) was used to induce local anesthesia, both at the mandible and at the upper left premaxilla. A double layer straight incision was first done below the mucogingival line in between the lower canines, to expose the apexes of the lower incisors as well as the symphysis area. The apexes of the lower incisors were then exposed; a surgical toilette was performed to remove the pathological tissue, followed by the creation of new apical seals obtained using a reinforced zinc-oxide cement (Bosworth® Super Eba*™*, Skokie, IL, USA) (Figure 4). Subsequently, an osteotomy was conducted with rotating instruments in the underlying mandibular symphysis area to harvest a corticocancellous bone block (Figure 5). The donor site was then filled with a native collagen sponge and a double layer suture was performed, with a 5-0 resorbable suture (Vicryl®, Ethicon Inc., Somerville, NJ, USA) on both the periosteum and the mucosal levels.

**Figure 6:**
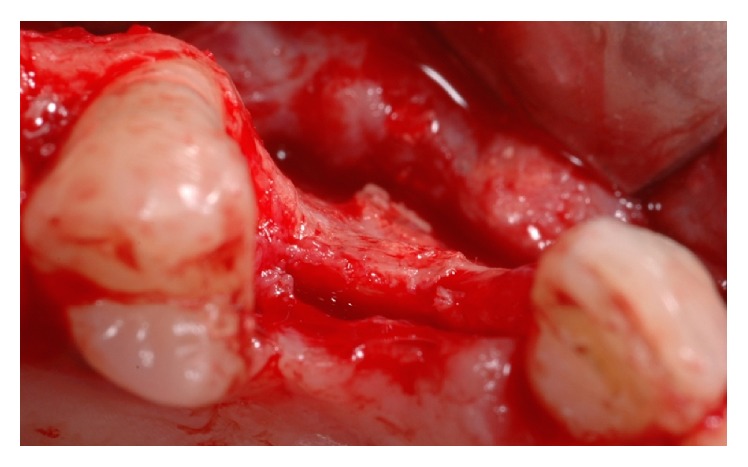
Anatomical view of the occlusal aspect of the edentulous ridge. The horizontal bone resorption is clearly represented.

**Figure 7:**
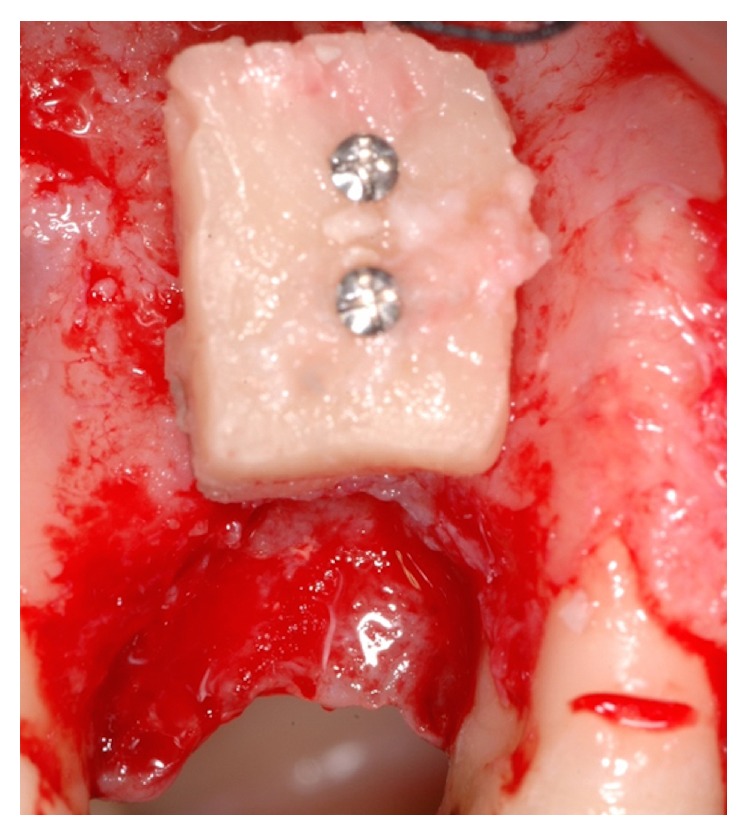
Bone block fixed to the vestibular cortex of the edentulous ridge by means of two transcortical screws.

**Figure 8:**
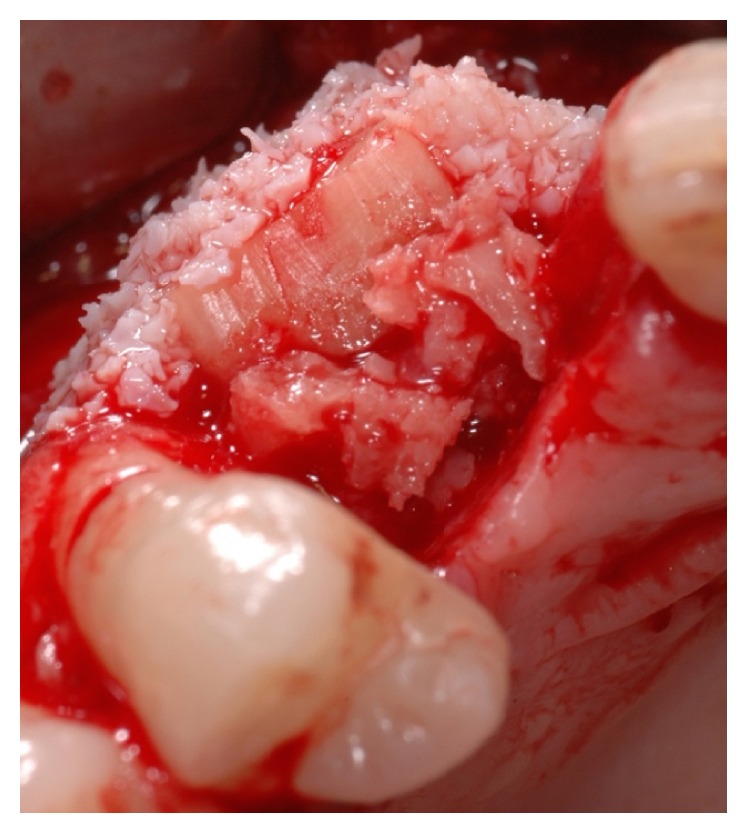
Particulated anorganic bovine bone placed on top of the bone block to reduce the physiological shrinkage of the graft due to bone remodelling.

**Figure 9:**
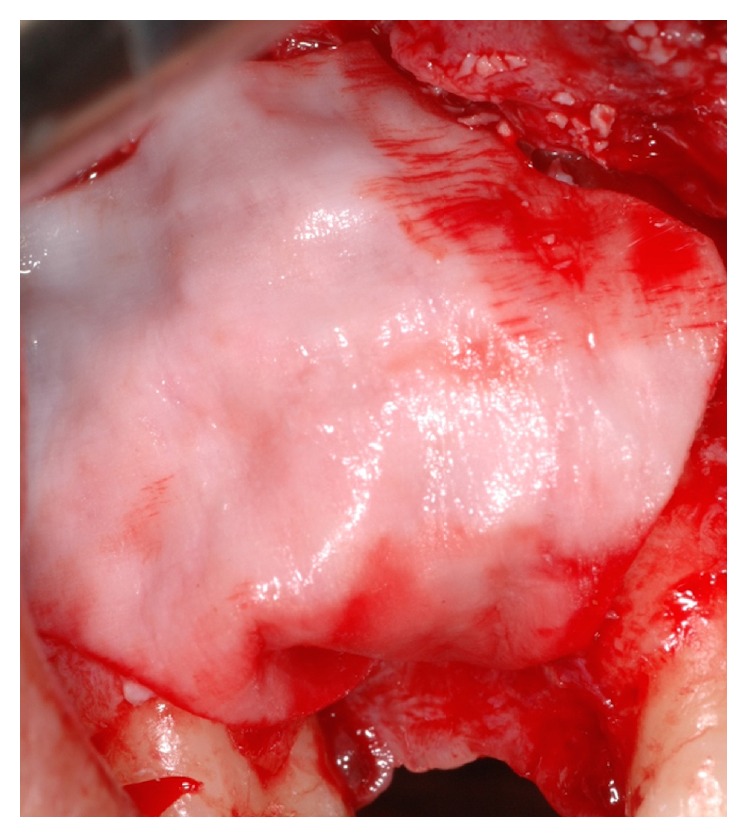
Resorbable collagen membrane laid over the graft to prevent soft tissue cells ingrowth.

The recipient site was then prepared with a trapezoidal full thickness flap from the mesial side of the right lateral incisor up to the distal side of the right first bicuspid. The bone was then exposed (Figure 6) and the cortical plate was perforated with a round bur under copious irrigation with sterile physiological saline solution to promote rapid angiogenesis and migration of osteogenic potential cells from the endosteal compartment. The block, previously shaped, was adapted to the recipient site and fixed to the residual ridge with two transcortical screws (Figure 7). The edges of the block were then smoothened with an oval bur and the graft was covered with a thin layer of anorganic bovine bone granules (Bio-Oss®, Geistlich Pharma AG, Wolhusen, Switzerland) (Figure 8) and a collagen membrane (Bio-Gide®, Geistlich Pharma AG, Wolhusen, Switzerland) (Figure 9). Flaps were released with sharp dissection to allow tension-free closure. Horizontal mattress and single stitches were used to seal the surgical wound.

**Figure 10:**
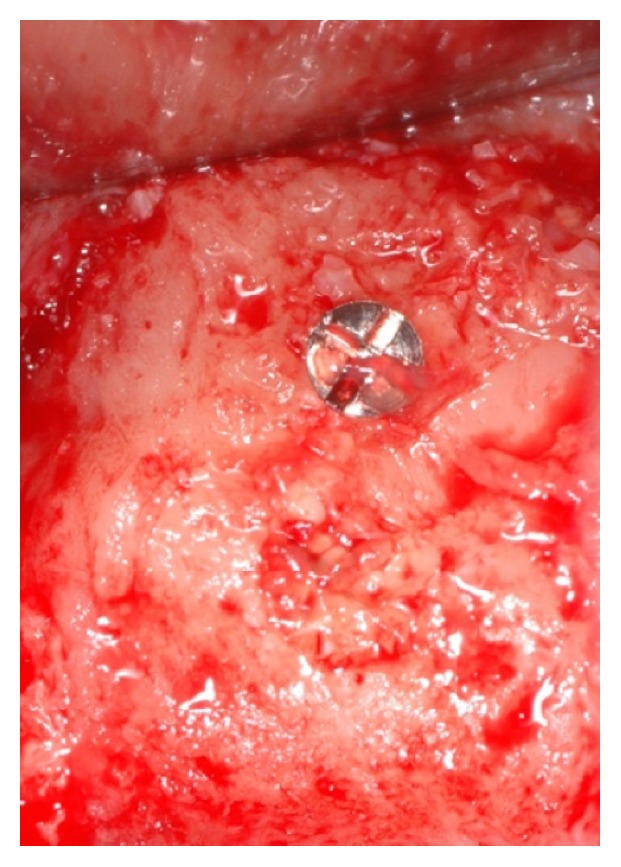
Clinical view of the bone block during the reentry procedure. The graft is completely integrated with the surrounding bone, with no volumetric resorption as demonstrated by the bone levels circumferentially around the transcortical screw.

**Figure 11:**
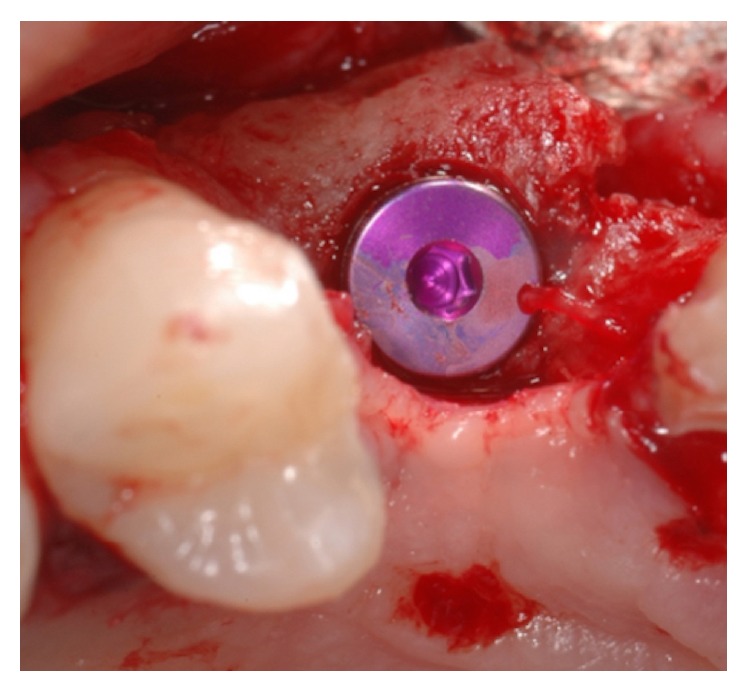
Dental implant inserted in a prosthetically guided position in augmented bone.

**Figure 12:**
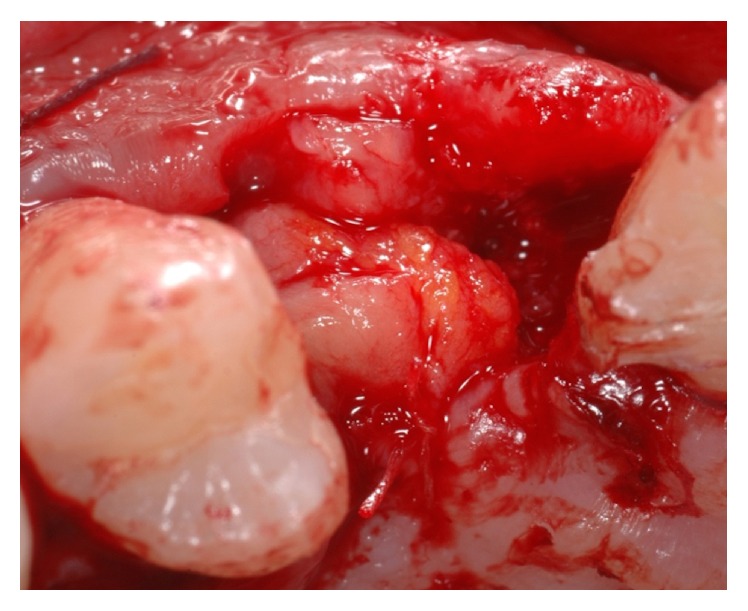
Connective tissue graft harvested from the palatal mucosa of the flap to increase soft tissue thickness.

The reentry procedure was accomplished after a healing period of four months. The healing proceeded uneventfully and no complications were encountered. After elevation of a mucoperiosteal flap, no signs of graft resorption were observed as from the absence of exposed threads of the transcortical screws (Figure 10). A 4.3 mm diameter per 13 mm length single implant (Camlog Screw-Line, Camlog Biotechnologies, Basel, Switzerland) was therefore placed in a prosthetically guided position (Figure 11). A connective tissue graft (CTG), harvested from the inner part of the palatal mucosa at the surgical site, was placed to increase the thickness of the soft tissues (Figure 12).

**Figure 13:**
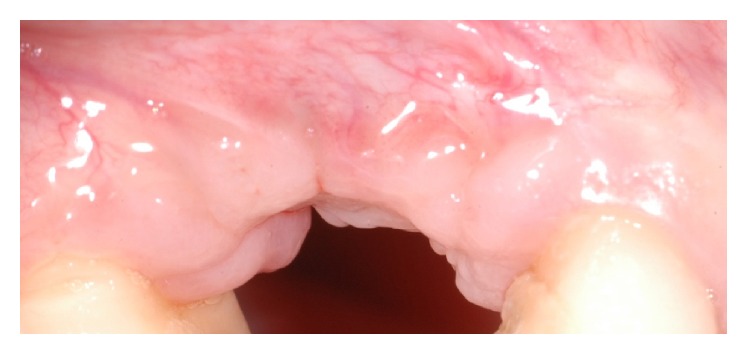
Soft tissues defect after 4 months of healing.

**Figure 14:**
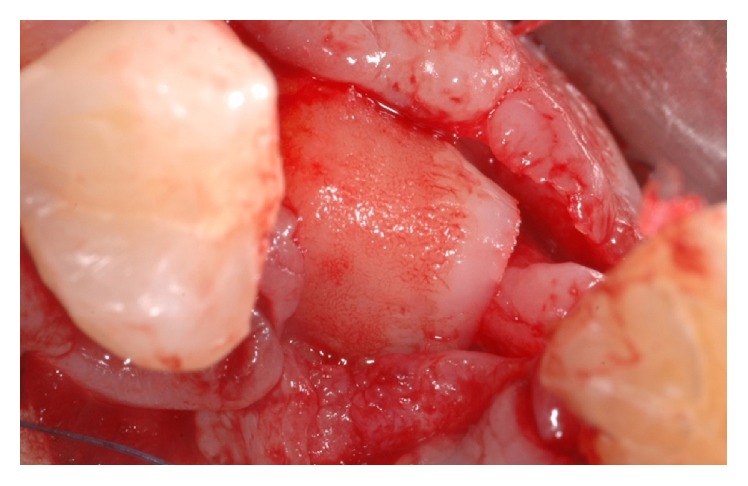
A free deepithelized gingival graft was used to enhance the amount of peri-implant soft tissues.

**Figure 15:**
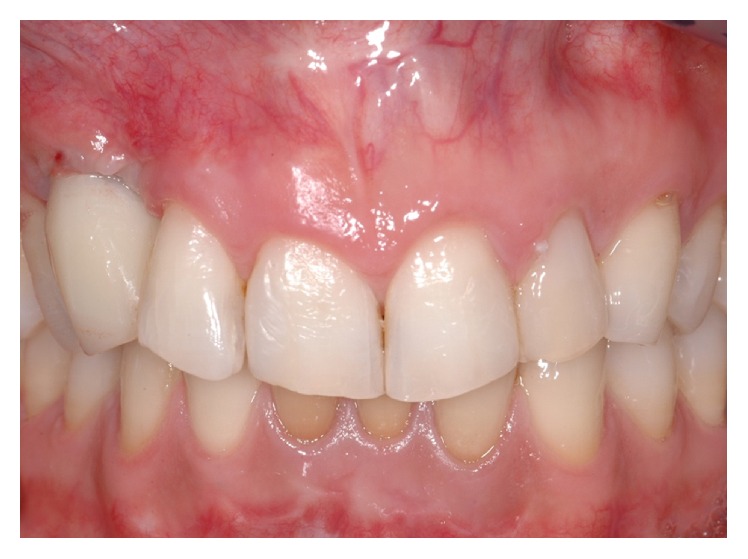
First provisional crown in situ. After a 2-month healing time, the soft tissue profile was still unaesthetic when compared to the contralateral side.

After 4 months, being the quality of the soft tissues unsatisfactory (Figure 13), a free deepithelized gingival graft was used to enhance the amount of tissue (Figure 14). A first provisional crown was connected to the implant two months later, but the coronal level of the soft tissues was still aesthetically unacceptable, when compared to the contralateral canine (Figure 15).

**Figure 16:**
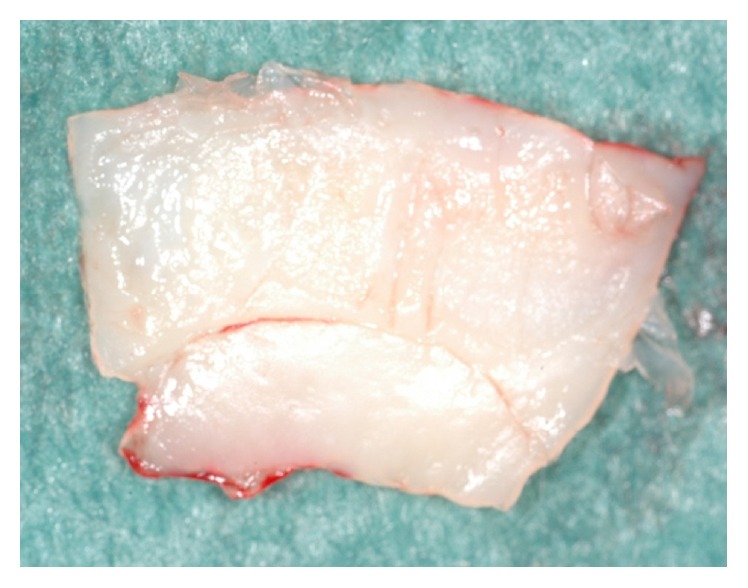
Detail of the epithelialized (lower part) and subepithelial (upper part) connective tissue graft.

**Figure 17:**
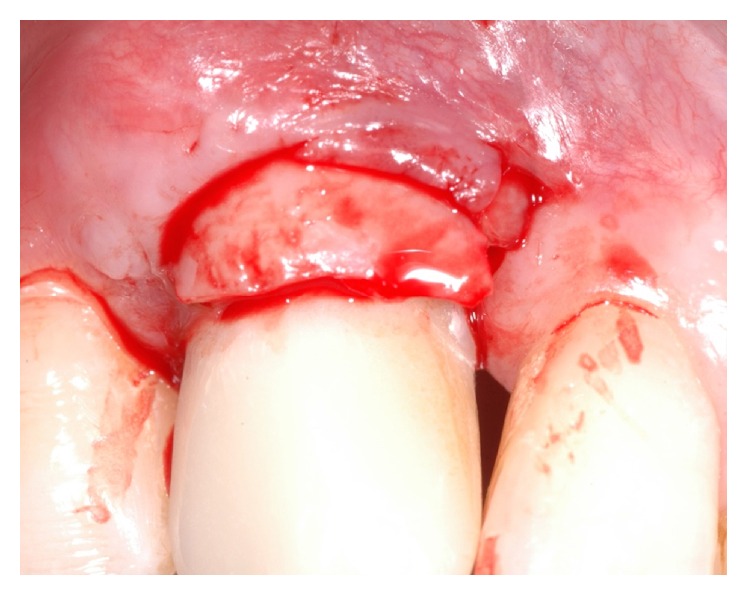
Detail of the epithelial half-moon coronal portion of the graft facing toward the prosthetic crown.

**Figure 18:**
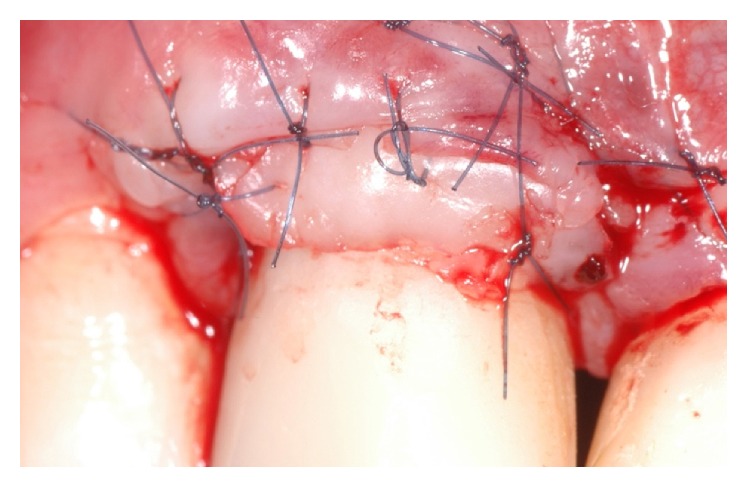
Combined soft tissue graft in situ.

**Figure 19:**
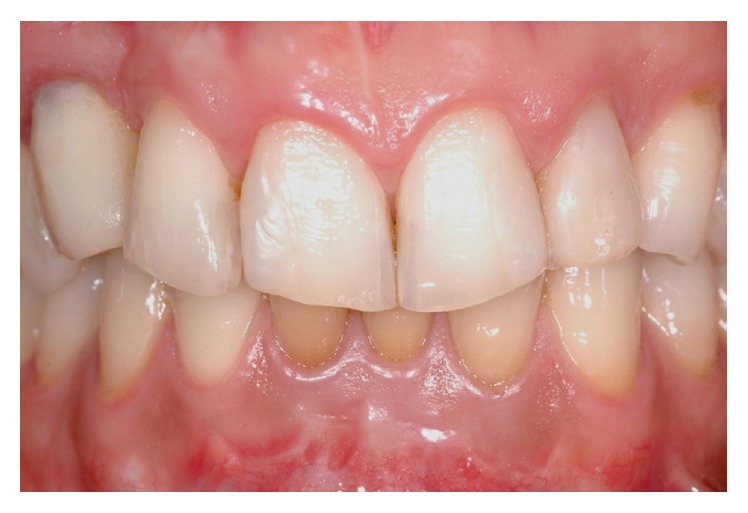
New temporary crown placed and adapted to the recipient site.

**Figure 20:**
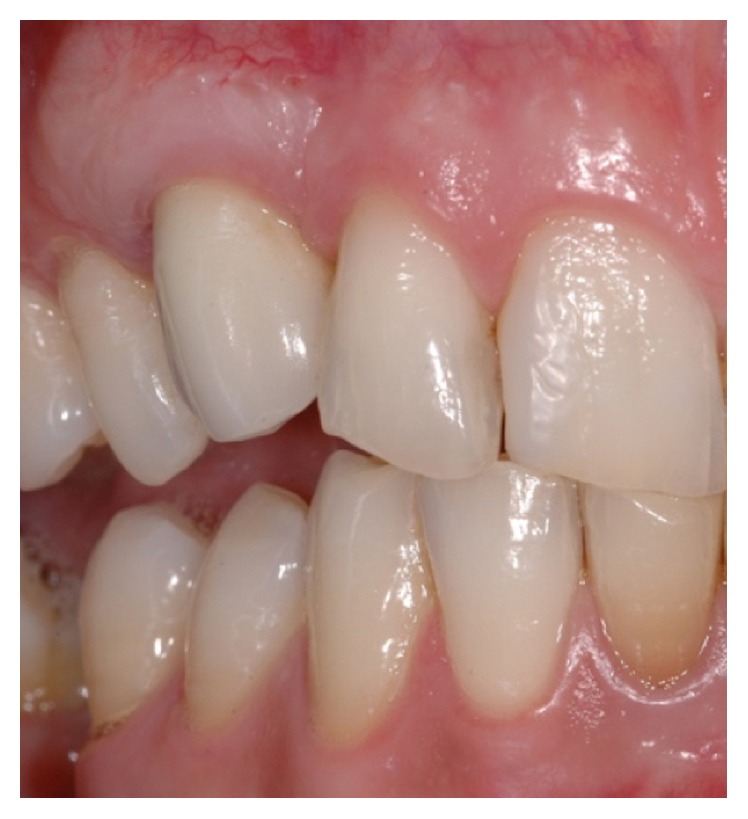
Final gold ceramic crown placed after 6 months of healing.

Hence, the need to move the gingiva more coronally induced the clinician to detect a technique able to correct the difference in height between the two canines. Being it impossible to perform a coronally repositioned flap, due to the presence of the acrylic crown associated with the absence of enough keratinized tissue, a combination of epithelialized and subepithelial CTG was chosen. A free gingival graft (FGG) was therefore harvested from the premolar-molar region of the palatal vault, prepared so as the apical part was disepithelialized leaving the connective tissue exposed, whereas the coronal part corresponding to the portion of the crown to be covered was left epithelized (Figure 16). The recipient site was then prepared with a 64C beaver blade to create a partial thickness envelope around the canine gingival margin. Subsequently, the upper part of the graft consisting of connective tissue was inserted by leaving out the epithelial half-moon coronal portion (Figure 17). A 6-0 nylon suture (Ethilon®, Ethicon Inc., Somerville, NJ, USA) was used to secure the graft in the proper position (Figure 18). A new temporary crown was placed and adapted to the recipient site (Figure 19). Six months later, impressions were taken and the final gold ceramic crown was placed (Figure 20).

**Figure 21:**
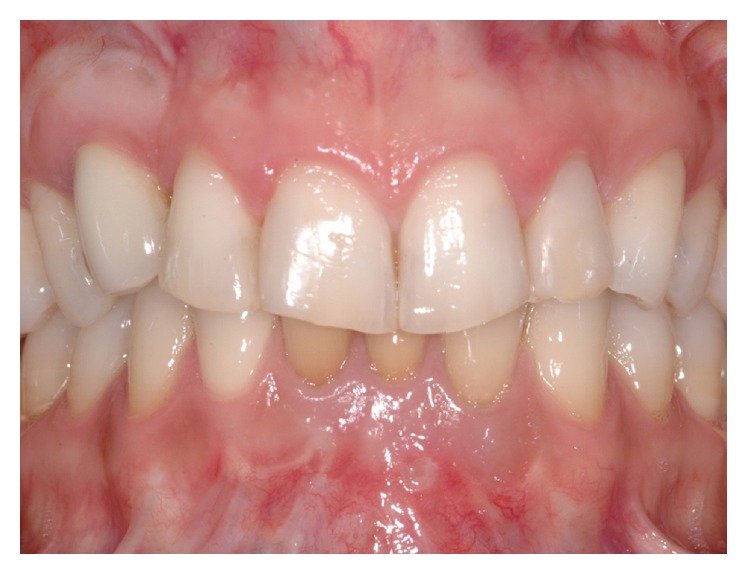
Three-year clinical follow-up demonstrating the volumetric stability of the soft tissue graft and the acceptable aesthetic result compared to the contralateral side.

**Figure 22:**
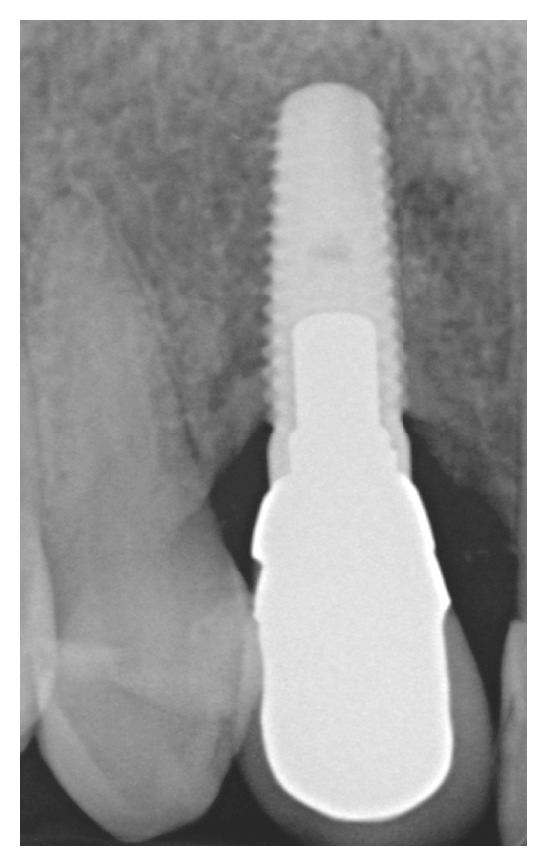
Three-year periapical X-ray. The implant appeared well integrated and the mesial and distal bone levels showed a physiological remodelling.

At the recall visit three years after the delivery of the final prosthesis, gingival levels of the upper canines appeared almost symmetric and clinically stable, with < 3 mm probing depths and no bleeding on probing circumferentially around (Figure 21). The radiological examination conducted with a periapical X-ray using the long-cone paralleling technique demonstrated the maintenance of bone levels at the mesial and distal aspect of the implant (Figure 22).

## 3. Discussion

The present case report demonstrated the volume maintenance of hard and soft tissue autografts in a critical anatomical situation characterized by ridge atrophy and thin biotype in an aesthetic zone.

Alveolar bone grafting with autogenous mandibular bone has shown excellent survival and success rate of implants and a low tendency to long-term surface resorption, as well as good patients' tolerance with minimal side effects [2]. Based on current findings, the surface resorption of ramus and symphyseal blocks grafted in the anterior maxilla is similar; however corticocancellous blocks might be retrieved more effectively and with a higher amount from the chin [3]. Besides these considerations, the need for a second surgical site in the symphyseal region was another factor addressing the clinician to consider the chin as a donor site. To prevent significant graft resorption during the integration phase, anorganic bovine bone granules have been placed over the block and covered with a bioabsorbable collagen membrane. At the reentry surgery, the transcortical screws were entirely surrounded by remodeled bone, meaning that no graft resorption occurred during the healing time. This result favorably comply with those reported by Maiorana et al., indicating that bovine bone particles might prevent excessive bone remodelling due to its osteoconductive properties [4]. The stability of the mesial and distal bone levels has been radiographically documented at the 3-year follow-up examination, demonstrating the short-term reliability of the surgical technique even in a thin biotype patient. Indeed, thin tissue biotype is associated with an increased risk for unfavorable treatment outcomes following surgical interventions, due to increased friability, impaired vascularization, and thinner underlying bone [5]. The present result corroborates the finding that a thin periodontal biotype does not significantly affect the volume integrity of autogenous bone blocks harvested from the mandibular symphysis and transplanted in the premaxilla for implant placement purposes [6].

On the other hand, thin tissue biotype might be associated with a lack of keratinized mucosa and increased risk of mucosal recession together with unsatisfactory aesthetic outcomes [5]. Hence, several techniques have been advocated to increase keratinized tissue surrounding implants, including FGG and CTG. Nevertheless, recent findings pointed out the fact that periodontal plastic surgery procedures around dental implants gave good initial results from the inflammation involved in wound healing, but virtually all cases resulted in some significant recession as healing resolved and the tissue matured [7]. As a matter of fact, in the present report, both CTG and FGG showed a certain tendency to relapse in a short-term period. This might be related to the anatomical differences existing between teeth and implants, including the lack of periodontal ligament and therefore a decreased vascular supply for the graft. Such resorption rate prompted the use of combined epithelialized-subepithelial grafts for augmenting the keratinized mucosa around implants. The rationale is based on an increased vascularization provided by the connective tissue portion, which is sutured inside the partial thickness envelope so as to receive a flow of plasma and ingrowth of capillaries from the surrounding connective tissue. Consequently, as confirmed by the present case report, this technique might be able to decrease the potential partial shrinkage of the graft due to lack of blood supply, reducing the failure and dehiscence rate [8, 9].

In conclusion, when autogenous sources and biomaterials are coupled together, their synergistic potential is able to provide a successful result even in more demanding areas such as the aesthetic zone of the premaxilla.
